# Salinity tolerance mechanisms in glycophytes: An overview with the central focus on rice plants

**DOI:** 10.1186/1939-8433-5-11

**Published:** 2012-06-22

**Authors:** Tomoaki Horie, Ichirou Karahara, Maki Katsuhara

**Affiliations:** 1 Division of Applied Biology, Faculty of Textile Science and Technology, Shinshu University, 3-15-1, Tokida, Ueda, Nagano, 386-8567 Japan; 2 Department of Biology, Graduate School of Science and Engineering, University of Toyama, 3190 Gofuku, Toyama, 930-8555 Japan; 3 Institute of Plant Science and Resources, Okayama University, 20-1, Chuo-2-chome, Kurashiki, Okayama, 710-0046 Japan

## Abstract

Elevated Na^+^ levels in agricultural lands are increasingly becoming a serious threat to the world agriculture. Plants suffer osmotic and ionic stress under high salinity due to the salts accumulated at the outside of roots and those accumulated at the inside of the plant cells, respectively. Mechanisms of salinity tolerance in plants have been extensively studied and in the recent years these studies focus on the function of key enzymes and plant morphological traits. Here, we provide an updated overview of salt tolerant mechanisms in glycophytes with a particular interest in rice (*Oryza sativa*) plants. Protective mechanisms that prevent water loss due to the increased osmotic pressure, the development of Na^+^ toxicity on essential cellular metabolisms, and the movement of ions via the apoplastic pathway (i.e. apoplastic barriers) are described here in detail.

**Electronic supplementary material:**

The online version of this article (doi:10.1186/1939-8433-5-11) contains supplementary material, which is available to authorized users.

## Background

The global climate change is feared to promote rapid soil degradations in agricultural lands worldwide. Soil salinization is one of the serious soil degradations, which can arise from natural causes and human-mediated activity such as irrigation in arid and semi-arid regions. Approximately 20% of the irrigated lands in the world are presumably affected by soil salinization (Yeo [1999]). Salinity stress significantly reduces growth and productivity of glycophytes, which are the majority of agricultural products. The term “salinity” represents all the problems of the soil accumulating excessive salts, which can be categorized into sodic (or alkaline) and saline soils (IRRI [2011]). Sodic soils having a poor soil structure generally spread over arid and semi-arid regions, retaining high concentrations of Na^+^ at the exchangeable site of clay particles in the soil, which shows high pH (greater than 8.5) with a high exchangeable sodium percentage (ESP > 15) (IRRI [2011]). Saline soils can be generally found in arid regions, estuaries, and coastal fringes, which are dominated by Na^+^ ions with electrical conductivity (EC) more than 4 dS/m that corresponds to approximately 40 mM NaCl (IRRI [2011]; Munns and Tester [2008]). Moreover, saline soils exhibit ESP of < 15 and much lower pH values than the sodic soils (IRRI [2011]).

**Figure 1:**
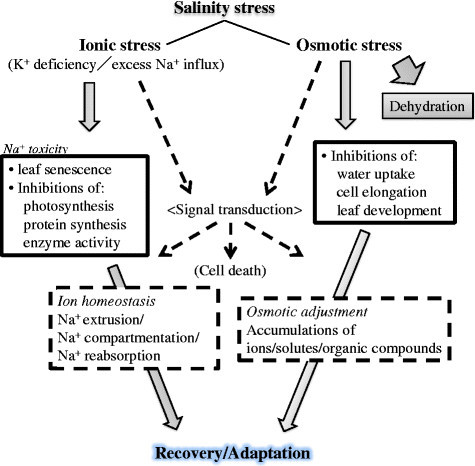
**A schematic summary of the stresses that plants suffer under high salinity growth condition and the corresponding responses that plants use in order to survive these detrimental effects.**

Plants have to cope with two major stresses under high salinity, osmotic stress and ionic stress (Figure 1). The former stress immediately comes over plants in accordance with a rise in salt levels outside the roots, which leads to inhibitions of water uptake, cell expansion and lateral bud development (Figure 1) (Munns and Tester [2008]). The latter stress phase develops later when toxic ions such as Na^+^ accumulate in excess in plants particularly in leaves over the threshold, which leads to an increase in leaf mortality with chlorosis and necrosis, and a decrease in the activity of essential cellular metabolisms including photosynthesis (Figure 1) (Yeo and Flowers [1986]; Glenn et al. [1999]). Recent molecular physiological and molecular genetic studies have increasingly gained knowledge for the protection mechanisms that plants use to cope with detrimental effects of salinity stress (Blumwald [2000]; Zhu [2002]; Pardo et al. [2006]; Munns and Tester [2008]; Horie et al. [2009]; Hauser and Horie [2010]). Many studies also highlight the significance and relevancy of the functions/regulations of important membrane proteins such as water channels and Na^+^ transporters (Horie and Schroeder [2004]; Maurel et al. [2008]; Ward et al. [2009]) and also signaling molecules (Zhu [2002]) to plant salt tolerance.

In this review, we summarize the problems caused by soil salinity and molecular mechanisms that protect plants from salinity stress, combining knowledge from classic physiology with the recent findings. Rice is the most salt sensitive among cereals (Munns and Tester [2008]). In rice, it has been observed that the rate of Na^+^ uptake into shoots mediated by the intrusive apoplastic ion transport is considerably high under salinity stress (Yeo et al. [1987]; Yadav et al. [1996]; Ochiai and Matoh [2002]). Therefore, in addition to the first two sections where responses of plants to osmotic stress and ionic stress are mentioned, we particularly highlight morphological traits/barriers of plant roots under salinity stress in the last section of this review. Current achievements of investigators and future prospects are discussed.

### 1. Responses to osmotic stress caused by high salinity

Salinity-induced osmotic stress reduces water uptake into plant roots. Plants regulate water transport under salinity stress because a sufficient amount of water is indispensable for the cells to maintain their growth and vital cellular functions such as photosynthesis and metabolisms. In the long distance water transport from roots to shoots, evaporation is one of the main motive forces for the water movement, especially in the apoplastic pathway. Salinity/osmotic stress directly (Yeo et al. [1985]) or indirectly via hormonal regulation (Jia et al. [2002]) induces a stomatal closure, which leads to a reduction in the evaporation and overall water transport. Along with the apoplastic pathway, symplastic and transcellular pathways are also important in water transport in plants. In these pathways, where the water is transported across the membrane, the water potential (Ψ) plays a central role in the driving force for the water movement. Although some theoretical issue regarding a biological cause of the water flux remains to be discussed (Kramer and Boyer [1985]), water flux is positively correlated with the product of water potential difference (ΔΨ) and hydraulic permeability (*L* p). In case of water uptake in root cells, ΔΨ is the difference between Ψ of extracellular solution and intracellular sap solution. Under non-stress condition, intracellular Ψ is generally more negative than that of the soil solution, resulting in water influx into roots according to the water potential gradient. The water potential (Ψ) is approximately consistent with the sum of the pressure potential (Ψ_p_) and the osmotic potential (Ψ_osm_, equivalent to the osmotic pressure of salt solution but with minus sign because they work in opposite direction).

**Figure 2:**
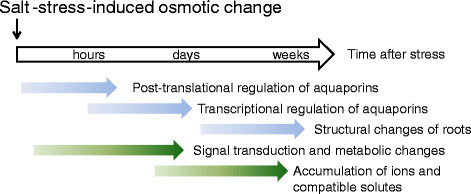
**Timeline of regulations and changes in symplastic water-related functions of a plant cell after salt-induced osmotic changes.** Blue arrows indicate functions included in the regulation of the water permeability (conductance) of roots, and green arrows indicate functions of the cellular osmotic adjustment.

Because of dissolved ions that decrease extracellular Ψ_osm_, salinity stress immediately reduces ΔΨ thus water influx. If the water potential gradient is reversed due to severe salinity/osmotic stress (that is, an excessive amount of dissolved ions decreases extracellular Ψ_osm_ remarkably), water efflux from roots (dehydration) can occur. To minimize the influence of a reduction in water influx or dehydration upon the growth under salinity/osmotic stress, plants set independent strategies in motion by regulating the root *L* p (*L* p_r_) and attempting to restore ΔΨ (Figure 2). Active regulation of intracellular Ψ_osm_, which re-establishes ΔΨ, can be achieved by accumulating solutes including organic compounds (for details, see below). However, time is required (> several hours or days) to accumulate enough solutes inside the cell to get a decrease in intracellular Ψ_osm_ (osmotic adjustments). Signal transduction and changes of related-gene expression, in contrast, are a relatively quick response (Figure 2). Fine-tuning of *L* p_r_ that occurs within hours is important for adaptive regulation under salinity/osmotic stress. Several days after the salinity/osmotic stress, the whole-root water conductance can also be regulated by increasing the total root surface area via changes in the root morphology because the whole-root water conductance is the product of *L* p_r_ by the total root surface area. For example, a stimulation of the lateral root formation was observed in *Arabidopsis thaliana* under mild salt stress (Zolla et al. [2010]). In rice, increased growth of roots in depth was found under drought stress (Asch et al. [2005]), but the detailed response of rice roots to drought or salinity stress is yet to be elucidated.

### Regulations of *L* p_r_and aquaporin water channels

Intracellular Ψ of cells with full turgor of maize (*Zea mays*) or wheat (*Triticum aestivum*) was reported to be in a range of 0.5-0.7 MPa (Munns, [2002]). Consistently, the corresponding Ψ of rice root cells was measured to be -0.5 MPa (Katsuhara, unpublished data). Osmotic Ψ of -0.5 MPa is equivalent to a 220 mOsm solution or a 124 mM NaCl solution, which means ΔΨ is reduced but not eliminated by salinity stress of 100 mM NaCl or less. Under such circumstances, an enhancement of *L* p_r_ might compensate the reduction of water influx. As a matter of fact, however, reductions in *L* p_r_ were recorded in several plant species including *Arabidopsis* and maize under salinity stress of less than 100 mM NaCl (Azaizeh and Steudle [1991]; Peyrano et al. [1997]; Carvajal et al. [1999]; Martínez-Ballesta et al. [2000]; Martínez-Ballesta et al. [2003]; Boursiac et al. [2005]). In roots of barley (*Hordeum vulgare*) seedlings, no significant change in the *L* p_r_ has been reported when plants were treated with 100 mM NaCl for 4 hrs in a sharp contrast to the severe *L* p_r_ repression by 200 mM NaCl treatments for 4 hrs (Horie et al. [2011b]). However, more detailed time course *L* p_r_ analyses using barley seedlings imposed by 100 mM NaCl stress demonstrated that severe *L* p_r_ repression occurs within 1 hr and the repression status lasts at least for 24 hrs with a complex temporal *L* p_r_ retrieval (Kaneko, Horie and Katsuhara, unpublished). Such *L* p_r_ reductions should be effective to prevent dehydration under stress conditions more severe than -0.5 MPa (i.e. equivalent to 124 mM or more of NaCl). Severe salinity stress markedly decreases Ψ of the soil solution, which can reverse the osmotic gradient between the inside and the outside of root cell, which generates water efflux (dehydration). In these conditions, shutdown of the water transport attributed to the *L* p_r_ reduction should be essential to minimize water loss at the initial phase of severe salt stress for survival (Kjellbom et al. [1999]; Hachez et al. [2006]; Horie et al. [2011b]). However, the reason why NaCl treatments with the concentration of less than 100 mM evokes significant reductions in *L* p_r_ in many plant species is yet to be clarified. Possible significances of the phenomenon would be: (i) Plants shut down *L* p_r_ even upon moderate salinity stress conditions to get ready for more severe stress in advance because such a sequence occurs in nature (that is, moderate stress gradually succeeds to more severe one); and (ii) *L* p_r_ reductions could be a sign of conversion of the growth status of plant cells from the rapid growth mode with high water absorption to the protect/tolerant one with less water uptake as a strategy for the survival under salinity stress (Horie et al. [2011b]).

Interestingly, no significant change in *L* p_r_ was observed in the plants of japonica rice cultivar Nipponbare under salinity stress of 100 mM NaCl within 24 hrs by the pressure chamber method (Kaneko and Katsuhara, unpublished data). The result suggests that at least Nipponbare rice plants might not be able to promote an immediate repression of the *L* p_r_ in response to the osmotic stress phase. Similar exceptions of no influence of salinity stress on *L* p_r_ have been reported using tobacco (Tyerman et al. [1989]) and relatively mature barley plants (Munns and Passioura [1984]). Whether the salinity/osmotic-induced *L* p_r_ down-regulation is an essential component of salt tolerant mechanisms in plants and how much influence the existence of the down-regulation has on plant salt tolerance are important questions to be addressed. Moreover, whether the pattern of regulation of *L* p_r_ upon salinity/osmotic stress could be changed depending on the growth stage of the plant species as has been seen in two independent studies using mature barley plants (Munns and Passioura [1984]) and barley seedlings (Horie et al. [2011b]) is also an interesting question to pursue.

Water transport across cellular membranes is mediated by water channel activity of proteins that belong to the major intrinsic protein (MIP) family called aquaporins (Tyerman et al. [1999]; Javot and Maurel [2002]; Chaumont et al. [2005]; Maurel et al. [2008]). Aquaporins are known to be a pore-forming membrane protein, which transport water and low-molecular weight neutral compounds (Tyerman et al. [2002]; Maurel et al. [2008]). The plasma membrane intrinsic proteins (PIPs) being divided into two phylogenic subgroups PIP1 and PIP2 are one of the four major subfamilies of plant aquaporins and the most abundant aquaporins in the plasma membrane (Maurel et al. [2008]). Genetic evidence using tobacco and *Arabidopsis* plants have indicated that PIP aquaporins mediate water uptake by roots and are a predominant component of the *L* p_r_ (Martre et al. [2002]; Siefritz et al. [2002]; Javot et al. [2003]). Furthermore, the residual *L* p_r_ of salt-stressed *Arabidopsis* and paprika plants have been shown to be resistant to mercury that is a potent inhibitor of aquaporin-mediated water transport, indicating that the down-regulated *L* p_r_ component consists of most likely PIP aquaporin activity in these conditions (Carvajal et al. [1999]; Martínez-Ballesta et al. [2003]). These earlier studies together point that uncovering regulatory mechanisms on aquaporins at the molecular level, including transcriptional and post-translational modifications, are indispensable to understand the physiological significance of *L* p_r_ down-regulation upon salinity/osmotic stress in plants.

Aquaporin genes were found to form a large gene family in several plant species including *Arabidopsis* and maize (Chaumont et al. [2005]). Salinity-induced down-regulation of most aquaporin transcripts has been observed in roots of salt-stressed *Arabidopsis* and maize plants (Maurel et al. [2008]), suggesting that a transcriptional regulation on aquaporin genes contributes to the *L* p_r_ down-regulation under salinity stress. In roots of barley seedlings, the accumulation of ten *HvPIP* transcripts from 100 mM NaCl-treated plants is similar to those from non-stress condition. However, 200 mM NaCl stress significantly down-regulated the level of six out of ten *HvPIP* transcripts (Horie et al. [2011b]). The importance of post-translational mechanisms such as protein phosphorylation/dephosphorylation and dynamic changes in subcellular localization via membrane internalization on aquaporin-mediated water transport in roots is being focused (Boursiac et al. [2005]; Boursiac et al. [2008]; Maurel et al. [2008]; Horie et al. [2011b]). In *Arabidopsis*, 100 mM NaCl treatments induced redistribution of PIPs from the plasma membrane to internal compartments, which could account for the rapid *L* p_r_ down-regulation in 100 mM NaCl-treated *Arabidopsis* roots (Boursiac et al. [2005]). More recent findings further demonstrated that salinity-induced rapid *L* p_r_ down-regulation and redistribution of PIPs to internal compartments are controlled by the salicylic acid-mediated accumulation of reactive oxygen species (ROS), which is stimulated by salinity stress (Boursiac et al. [2008]). Together, these findings suggest that mechanisms to regulate PIP aquaporins are one of key mechanisms used by plants to maintain proper *L* p_r_, therefore water homeostasis during salinity/osmotic stress.

**Figure 3:**
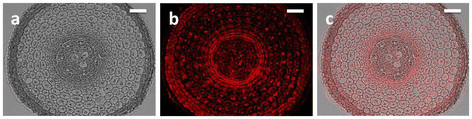
**Localizations of OsPIP2;1 in rice root tissues.** Rice plants were grown in a hydroponic culture solution and are placed in a growth chamber under a 12 h dark (20 C)/12 h light (25 C) photoperiod (370 μmol s^-1^ m^-2^) for 38 d. Tap root samples were fixed at 3 h after the onset of the light period. Root sections at around 4 mm from the root tip were subjected to immunocytochemistry using the anti-OsPIP2;1 antibody. **a,** Bright field image; **b,** fluorescent image; **c,** superimposed image of **a** and **b.** Bars represent 50 μm (unpublished, kindly provided by Dr. Junko Sakurai-Ishikawa of NARO Tohoku Agricultural Research Center, Japan).

In rice plants, 33 aquaporin genes including 11 *PIPs* were identified (Sakurai et al. [2005]). Recent works further showed the expression and localization patterns of PIP proteins, in which several OsPIP1 proteins, OsPIP2;1, and tonoplast-localized TIP proteins, OsTIP1;1 and OsTIP2;2, were found to express in both leaf blades and roots, while OsPIP2;3, OsPIP2;5 and OsTIP2;1 were expressed only in roots (Sakurai et al. [2008]; Sakurai-Ishikawa et al. [2011]). A ubiquitous localization of OsPIP2;1 with the abundance in the proximal end (close to the central cylinder) of the endodermis was observed (Figure 3, Sakurai unpublished data), which is similar to the expression pattern of OsPIP2;5 (Sakurai-Ishikawa et al. [2011]). These localizations imply important roles of OsPIP water channels in the water transport of rice plants. Although there is no report regarding post-translational regulations such as phosphorylation/dephosphorylation and endocytotic recycling on PIP aquaporins in rice under salinity stress, salinity/osmotic stress-induced reductions in expression levels of *OsPIP* and *OsTIP* mRNAs were detected in rice (Guo et al. [2006]; Li et al. [2008]). These results suggest that plant aquaporins could be involved in salt tolerant mechanisms by repressing the activity of water channels under salinity stress. In supporting to this hypothesis, over-expression of HvPIP2;1 from barley induced salt hypersensitivity phenotypes in transgenic rice plants (Katsuhara et al. [2003]). On the contrary, over-expression of some OsPIPs was found to enhance salt tolerance in *Arabidopsis* (Guo et al. [2006]) and rice (Kitagawa, personal communication). These complicated results might be attributed to the different functions of introduced aquaporin genes. Further investigations are needed to reveal the exact physiological roles of aquaporins in salinity/osmotic tolerance mechanisms *in planta*.

### Osmotic adjustments and compatible solutes

Osmotic adjustments by means of solute accumulations inside the cell are essential to reduce the cellular Ψ_osm_ against an osmotic gradient between root cells and outside saline solution, which eventually restore the water uptake into roots during salinity stress (Greenway and Munns [1980]). Ion accumulations in the cytosol (mainly K^+^) and in the vacuole (Na^+^, especially in salt tolerant cultivars/species) are also found to be important for the osmotic adjustment of plant cells (Gorham et al. [1985]). In addition to the accumulation of ions for cellular osmotic adjustment, certain organic compounds are known to accumulate in the cytosol under salinity/osmotic stress conditions. Such compounds are called compatible solutes (Bohnert and Shen [1999]). Compatible solutes were initially determined as compounds that are non-toxic even when they are highly accumulated in the cytosol and contribute to decrease the cytoplasmic water potential. In addition to the role in osmotic adjustments, compatible solutes seem to function as a chaperone protecting enzymes and membrane structures, and as a scavenger reducing radical oxygen species under stress conditions including salinity stress (Bohnert and Shen [1999]). Rice has two genes encoding the betaine aldehyde dehydrogenase, which catalyzes betaine aldehyde to glycine betaine (GB), a compatible solute. However, rice cannot synthesize GB because of the lack of an upstream enzyme, the choline monooxidase (CMO), which convert a choline to a betaine aldehyde. Introductions of spinach CMO genes or the *Arthrobacter pascens* choline oxidase into rice plants promoted the synthesis of GB in the transgenic rice plants (Sakamoto et al. [1998]; Shirasawa et al. [2006]). However, only relatively small amount of GB accumulation and slight enhancement of salt tolerance of transgenic rice plants were observed in some conditions tested, probably because of low activities and/or miss-localization of the introduced enzymes (Shirasawa et al. [2006]).

### 2. Na^+^ over-accumulation and components for the protection from Na^+^ toxicity under salinity stress

Over-accumulated Na^+^ in the cytoplasm during salinity stress develops toxicity and disturbs essential cellular metabolisms such as protein synthesis, enzyme activity and, in the case of cells that compose the source organ, photosynthesis (Yeo and Flowers [1986]; Glenn et al. [1999]; Tsugane et al. [1999]; Blaha et al. [2000]). At the whole plant level, salinity stress leads to Na^+^ over-accumulation in shoots particularly in old leaves, and many reports have suggested that restricting Na^+^ accumulation in shoots under salinity stress is associated with salt tolerance of wheat and barley (Jeschke [1984]; Gorham et al. [1990]; Munns and James [2003]; Garthwaite et al. [2005]). Moreover, it has been also reported that Na^+^ accumulation in shoots is relatively well correlated with the survival of rice plants under salinity stress (Yeo et al. [1990]). Ionic stress eventually triggers premature senescence of older leaves with stress symptoms such as chlorosis and necrosis (Munns [2002]; Munns et al. [2006]), which in turn significantly reduces growth and productivity of cereals. Therefore, effective strategies for glycophytes to cope with salinity stress are to keep cytosolic Na^+^ levels low at the cellular level and to keep shoot Na^+^ concentrations low at the whole plant level. In addition to these factors, acquisition and maintenance of K^+^ were found to have a considerable impact on plant salt tolerance (Wu et al. [1996]; Zhu et al. [1998]). Maintenance of high cytosolic K^+^/Na^+^ ratios especially in shoots have been strongly suggested to be crucial for salt tolerance of glycophyte plants (Gorham et al. [1987]; Gorham et al. [1990]; Blumwald [2000]; Ren et al. [2005]; Sunarpi et al. [2005]; Yamaguchi and Blumwald [2005]; Hauser and Horie [2010]). In fact, rice cultured cells overexpressing *OsKAT1* cDNA, which encodes a Shaker-type K^+^ channel, had enhanced cell growth in the presence of 100 mM and 200 mM NaCl (Obata et al. [2007]). OsKAT1-expressing cells accumulated more K^+^ during salinity stress, which resulted in higher K^+^/Na^+^ ratios of OsKAT1-expressing cells than control cells (Obata et al. [2007]). Furthermore, a more recent study demonstrated that tobacco cultured cells expressing the rice OsHAK5 transporter that exhibits relatively Na^+^ insensitive K^+^ uptake activity showed an enhancement of growth under salinity stress due to increases in K^+^ accumulations accompanied with decreases in Na^+^ accumulations as proved by the high K^+^/Na^+^ ratios of the cells (Horie et al. [2011a]). These findings further supported a positive impact of a stable K^+^ acquisition on the cellular salt tolerance.

The underlying mechanisms of Na^+^ entries into plant roots via both symplastic and apoplastic pathways are largely unknown. Based on the proposition by Yeo et al. [1987], at least four different entry mechanisms can be assumed: (i) ion channels/transporters that mediate Na^+^ selective transport at the plasma membrane of root epidermal/cortical cells, (ii) ion channels/transporters that mediate non-selective cation transport at the plasma membrane of root epidermal/cortical cells, (iii) Na^+^ intrusion into the root symplastic pathway due to a direct leakage through membrane bilayers or an injury in membrane bilayers, and (iv) a direct apoplastic intrusion into the xylem from the outside environment without biological selectivity.

During intrusive Na^+^ entries into the root, plants can exert “selectivity” at three independent biological membranes: the plasma membrane of epidermal/cortical cells, the tonoplast of cells in roots and shoots, and the plasma membrane of the xylem parenchyma cell. In this section, we focus on the physiological functions of several essential components that have been demonstrated to protect plants from Na^+^ toxicity on each biological membrane, with the special attention to the apoplastic Na^+^ flow, which is remarkably considerable in rice plants in the presence of high concentrations of Na^+^.

### A contribution of the apoplastic Na^+^ flow to the shoot Na^+^ accumulation in rice

A significance of the apoplastic space for the nutrition of higher plants in addition to the symplastic and transcellular solute transports, mediated by plasma membrane-localized channels/transporters and plasmodesmata, has been suggested (Sattelmacher et al. [1998]). It has been shown that a significant amount of Na^+^ transported to the shoots during salinity stress is through the apoplastic pathway (the so-called “bypass flow”) in the case of the rice plant, which is the most salt sensitive species among the cereals (Yeo et al. [1987]; Yadav et al. [1996]; Ochiai and Matoh [2002]; Anil et al. [2005]; Krishnamurthy et al. [2009]). Interestingly, in the presence of 100 mM NaCl, the upward Na^+^ transport rate in barley, which is the most salt tolerant cereal, is much lower (only 20%) when compared to that in rice plants (Munns [1985]), suggesting a significant contribution of Na^+^ bypass flow in salinity-induced shoot Na^+^ accumulation in rice plants. In roots, there are morphological components to prevent non-selective apoplastic flow of water and ions into the stele. These morphological components are Casparian bands and suberin lamellae at the root exo- and endodermis (Enstone et al. [2003]). Casparian bands and suberin lamellae are deposited in anticlinal walls and on the inner face of the primary cell walls, respectively. Though the mechanism of bypass flow has not been completely understood, bypass flow-mediated Na^+^ over-accumulation in shoots of rice plants is believed to be the outcome of a passive leakage of Na^+^ into the xylem over the morphological barriers. Since the apoplastic space of the leaf is relatively small, the effect of a large quantity of Na^+^ reaching the xylem in saline conditions is significant. In other words, the accumulation of even only a small portion of Na^+^ in the leaf apoplastic space causes large changes in ion concentrations of the space. According to the estimate of Yeo and Flowers ([1986]), even if 99% of arriving Na^+^ is successfully sequestered into the expanded rice leaves during salinity stress, the apoplastic Na^+^ concentration could reach 500 mM within 7 days, which would lead to severe cell dehydration and stomatal closure. Furthermore, shoot apoplastic Na^+^ accumulations were found to be negatively correlated with the survival of rice varieties including a highly salt tolerant cultivar Pokkali (Krishnamurthy et al. [2009]; Krishnamurthy et al. [2011]). Therefore, reducing Na^+^ transport to the shoots via apoplastic bypass flow is one of the primary subjects to solve in order to enhance salinity tolerance of rice plants.

**Figure 4:**
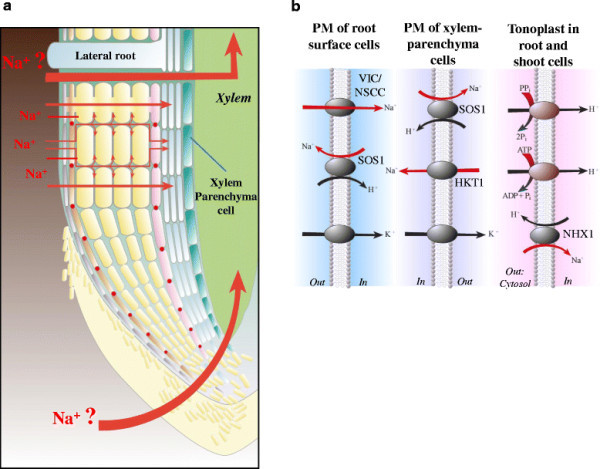
**Schematic summaries of Na**^**+**^** influx pathways into saline roots and primary protective mechanisms mediated by Na**^**+**^** transporters on important biological membranes. (a)** Schematic representations of several entries for Na^+^ influx into roots including cell-to-cell and apoplastic pathways. Thick red arrows represent hypothetical Na^+^ entry sites for the apoplastic bypass flow (see text). **(b)** Schematic representations of Na^+^ extrusion at the plasma membrane (PM) of the soil-cell interface (left), Na^+^ reabsorption at the PM of the xylem parenchyma cell (XPC: middle), and Na^+^ sequestration into the vacuole at the tonoplast (right). K^+^ transporting proteins in PMs of the soil-cell interface and the XPC respectively represent high-affinity K^+^ uptake channels/transporters and K^+^ efflux channels, which could couple with the each Na^+^ transporting mechanism in the PM.

Despite respectable efforts, the precise entry site for Na^+^ bypass flow remains to be determined. Due to the nature of apoplastic bypass flow, locations in roots with injuries and weak barrier areas were expected to be the potential entry sites. These include lateral root emerging sites and cell walls near the root apices (Yeo et al. [1987]) (Figure 4a). In monocot plants, lateral roots initiate to emerge at the pericycle near the phloem, disrupt the endodermal Casparian bands, and eventually break through the barrier in the exodermis as they develop (Ranathunge et al. [2005]). It was observed that Casparian bands and suberin lamella in both exo- and endodermis are undetectable at the root tip region (Ranathunge et al. [2003]; Schreiber et al. [2005]), suggesting an immature barrier status of the rice root tip. Recently, Faiyue et al. ([2010]) have reported that bypass flow was significantly increased in two independent lateral rootless mutants, *lrt1* and *lrt2*, and a crown rootless mutant, *crt1*, using an apoplastic tracer dye, trisodium-8-hydroxy-1,3,6-pyrenetrisulphonic acid (PTS). Based on their results, they concluded that the lateral root emergence does not contribute to be the entry site for the Na^+^ bypass flow (Faiyue et al. [2010]). In contrast, however, a more recent study has indicated the leakage of the tracer PTS into the primary root through the breaks created by lateral root emergences in both a salt sensitive cultivar IR20 and Pokkali plants (Krishnamurthy et al. [2011]). On the other hand, Ochiai and Matoh ([2002]) indicated that the tracer dye Fluostain I (also known as Calcoflour White M2R New) was intruded into the xylem with the strong fluorescence around the rice root tip region as well in the presence of 100 mM NaCl, suggesting a significant role of the root apical region in triggering bypass flow. It appears that the issue of the Na^+^ entry site for bypass flow still remains to be an open question. Further studies are needed to elucidate the locations for the Na^+^ entry in the apoplastic flow and the cause of shoot Na^+^ accumulation during salinity stress in rice plants. For more details of the apoplastic water and solute flow in plants, see the section below.

### Components for the Na^+^ extrusion from saline roots

Electrochemical analyses using barley and corn have predicted that active Na^+^ extrusion across the plasma membrane occurs under high salinity (Jeschke [1984]). Interestingly, the external K^+^-dependent net Na^+^ extrusion from Na^+^-loaded roots (K^+^-Na^+^ exchange) were found in cereals such as barley, wheat and rye, but not in other plant species such as onion, saltbush and buckwheat (Jeschke [1984]). This Na^+^ extrusion system, however, seems not to depend on the plasma membrane Na^+^/K^+^-ATPase that mediates the efflux 3Na^+^ and the influx 2 K^+^, which is ubiquitous in animal cells (Jeschke [1984]). Rather, the whole system were suggested to be composed of several independent activities in the plasma membrane, H^+^-pump ATPases, H^+^-Na^+^ antiport and the high-affinity K^+^ uptake (Jeschke [1984]), suggesting that the H^+^-driven Na^+^ exclusion is coupled to the high-affinity K^+^ uptake with the unknown mechanism in cereals (Figure 4b).

Three independent salt overly sensitive SOS mutant loci have been identified in the model plant *Arabidopsis thaliana* by a genetic screen, which render plants hypersensitive to high concentrations of Na^+^ and Li^+^ but not to the general osmotic stress (Zhu et al. [1998]). The initial genetic and physiological analyses revealed growth deficiency of *sos* mutants under low K^+^ conditions, which leads to an assumption that *sos* mutant loci are essential components for K^+^ acquisition and signal transduction during salinity stress (Wu et al. [1996]; Zhu et al. [1998]). In particular, *sos1* plants were demonstrated to exhibit deficiency in the high-affinity K^+^ uptake in roots, which suggested a primary role of the *SOS1* locus in mediating high-affinity K^+^ absorption into roots (Wu et al. [1996]). Interestingly, however, the *SOS1* gene was found to encode the plasma membrane-localized Na^+^/H^+^ antiporter, which extrudes Na^+^ out of the cell (Shi et al. [2000]). *SOS2* and *SOS3* genes were found to encode a protein kinase and a Ca^2+^ binding protein, respectively (Liu and Zhu [1997]; [1998]; Halfter et al. [2000]). They are later grouped into large protein families of calcineurin B-like proteins (CBL) and CBL-interacting protein kinases (CIPK), and therefore SOS2 and SOS3 are also known as CIPK24 and CBL4, respectively (Kolukisaoglu et al. [2004]). The working model of essential SOS components were proposed in which the SOS2/SOS3 complex targeted to the plasma membrane via N-myristoylation of SOS3 phosphorylates the SOS1 transporter to exclude Na^+^ (Qiu et al. [2002]; Quintero et al. [2002]) (Figure 4b). Further physiological analyses together with the *SOS1* promoter-GUS analyses revealed that *sos1* plants over-accumulate Na^+^ in both xylem sap and shoots, and that the physiological function of the SOS1 transporter is in Na^+^ extrusion particularly in the root apex and also in the long distant Na^+^ transport through the xylem vessel during salinity stress (Shi et al. [2002]) (Figure 4b). Note that an electrophysiological study later showed evidence that SOS1-dependent Na^+^ extrusion is in action in more matured epidermal cells as well (Shabala et al. [2005]). For more details, see a review (Zhu [2002]). Taken together, a significant reduction in the high-affinity K^+^ transport activity due to dysfunctional mutations in the *SOS1* gene in *Arabidopsis* plants implicates a complex interaction between high-affinity K^+^ uptake and Na^+^ extrusion in roots as has been observed in roots of cereals (see above), which remains to be elucidated.

A recent genetic study using barley on Zn^2+^ accumulation identified a candidate locus named *HvNax4*, which happened to have a considerable influence on the shoot Na^+^ accumulation (Lonergan et al. [2009]). The *HvNax4* locus was narrowed down to an approximately 200 kb region of the long arm of barley chromosome 1 H, and the *SOS3* gene homolog in barley, *HvCBL4*, in the region was found to co-segregate with the *HvNax4* locus (Rivandi et al. [2011]). It has been shown that rice plants also maintain large CBL and CIPK protein families similar to *Arabidopsis* plants (Kolukisaoglu et al. [2004]). Furthermore, the cDNAs encoding OsSOS1, OsSOS2 (OsCIPK24) and OsSOS3 (OsCBL4) have been isolated and the functions of those OsSOS proteins were investigated in yeast cells and in *Arabidopsis* plants (Martínez-Atienza et al. [2007]). The results demonstrated that all OsSOS proteins could coordinately function with AtSOS proteins in yeast cells and nicely complemented mutations in the corresponding *sos* mutant of *Arabidopsis* plants (Martínez-Atienza et al. [2007]). Together, these results suggest that the SOS-like salinity tolerance mechanism seems also to be conserved in barley and rice plants. However, important questions remain to be investigated: (i) why the high-affinity K^+^ uptake activity is significantly reduced in *sos1 Arabidopsis* mutants? (ii) whether the SOS1-type Na^+^/H^+^ exchanger exists and plays a primary function in salt tolerant mechanism in barley as in *Arabidopsis*?, (iii) if so, whether the SOS-like mechanism is a component constituting the high-affinity K^+^ uptake-coupled Na^+^ extrusion system in barley roots?, and (iv) whether rice roots exhibit activity of the high-affinity K^+^ uptake-coupled Na^+^ extrusion as found in other cereals, in which the OsSOS1-3 protein are involved?

### Components for the Na^+^ sequestration into the vacuole

Under salinity stress, it is crucial for plant cells to maintain the low cytosolic Na^+^ level while keeping the high level of K^+^, resulting in a high cytosolic K^+^/Na^+^ ratio that is preferable for vital cellular metabolisms (Jeschke [1984]; Blumwald [2000]). It has been shown that the sensitivity of cytosolic enzymes from glycophytes and halophytes to increased salt levels is comparable, suggesting that keeping a high cytosolic K^+^/Na^+^ ratio is a basic requirements for plant cells under salinity stress irrespective of the innate difference in the salt sensitivity (Glenn et al. [1999]). The sequestration of Na^+^ into vacuoles is an efficient mechanism to reduce the cytosolic Na^+^ concentrations (Yamaguchi and Blumwald [2005]). Furthermore, the sequestered Na^+^ ions in the vacuoles can in turn behave as an osmoticum, which helps in maintaining the osmotic driving force by promoting water uptake in saline environments.

Biochemical evidence of Na^+^/H^+^ antiport activity at the tonoplast membrane was reported in sugar beet (Blumwald and Poole [1985]; Blumwald and Poole [1987]). The molecular identity of the vacuolar Na^+^/H^+^ exchanger of *A. thaliana* plants, AtNHX1, was identified based on the similarity search for the *NHX1* gene of *Saccharomyces cerevisiae* on *A. thaliana* genome sequence (Apse et al. [1999]; Gaxiola et al. [1999]) (Figure 4b). Apse et al. ([1999]) indicated that *AtNHX1*-overexpression conferred increased salinity tolerance to the transgenic *Arabidopsis* plants, which were able to grown in the presence of 200 mM NaCl. This result demonstrates that vacuolar Na^+^/H^+^ antiport activity is indeed crucial for plant salt tolerance. *NHX* gene families have been identified in different plant species including cereals such as wheat, barley and rice since *AtNHX1* was isolated (Pardo et al. [2006]; Rodríguez-Rosales et al. [2009]; Yamaguchi et al. [2999]). Transgenic plants expressing various NHX1 transporters showed increased salt tolerance (For details, see a review: Yamaguchi and Blumwald [2005]), supporting the essentiality of Na^+^ sequestration in salt tolerance (Apse et al. [1999]).

In *Arabidopsis*, six *AtNHX* genes were identified, whose gene products can be divided into two classes, class I (AtNHX1-4) and class II (AtNHX5 and 6) (Pardo et al. [2006]). All class I transporters characterized to date are targeted to the tonoplast (Pardo et al. [2006]). The SOS2/CIPK24 protein kinase has been found to involve the regulation of class I NHX-mediated tonoplast Na^+^/H^+^ exchange in *Arabidopsis* (Qiu et al. [2004]). Tonoplast vesicles from *sos2* mutant plants but not from either *sos1* or *sos3* mutant plants exhibited a significant reduction in the Na^+^/H^+^ exchange activity (Qiu et al. [2004]), suggesting that SOS2/CIPK24 also functions in vacuolar Na^+^ sequestration presumably with some CBL(s) other than SOS3/CBL4. Unlike vacuolar-localized class I transporters, AtNHX5 and 6 class II transporters were postulated to function in endosomal vesicles (Pardo et al. [2006]). A recent study demonstrated that AtNHX5 and AtNHX6 indeed localize at endosomal compartments associated with Golgi and the *trans*-Golgi network (TGN) and loss of functions of both transporters rendered the *atnhx5 atnhx6* double mutant plants more salt sensitive (Bassil et al. [2011]). Moreover, the *atnhx5 atnhx6* plants exhibited reduced growth phenotypes even under normal growth condition. These phenotypes include decreases in leaf cell size and number in addition to the impairment of vesicular trafficking to the vacuole (Bassil et al. [2011]). These findings suggest that class II NHX-mediated ion homeostasis in endosomal compartments affects protein trafficking from the Golgi/TGN to the vacuole, which could cause increased sensitivity to salinity due to the deficiency in supply of essential components such as AtNHX1 into vacuoles when plants face salinity stress (Bassil et al. [2011]). For more details of NHX proteins, see reviews: (Pardo et al. [2006]; Rodríguez-Rosales et al. [2009]; Yamaguchi et al. [2999].

The vacuolar Na^+^/H^+^ exchange activity is driven by the vacuolar proton gradient established by two independent proton pumps, vacuolar H^+^-ATPase (V-ATPase) and vacuolar H^+^-translocating pyrophosphatase (V-PPase) (Blumwald [1987]) (Figure 4b). Constitutive overexpression of a V-PPase of *Arabidopsis*, AVP1, conferred increased resistance to high concentrations of Na^+^ and drought stress by enhancing cation uptake into vacuoles (Gaxiola et al. [1999]; Gaxiola et al. [2001]). These findings indicate that enhancement of the driving force for vacuolar Na^+^/H^+^ exchange activity is an efficient strategy to increase salinity tolerance in plants, and that vacuolar Na^+^ sequestration is crucial for plant salt tolerance.

Earlier biochemical analyses provided evidence that salt-tolerant *Plantago maritima* plants maintains a greater salt-induced Na^+^/H^+^ antiport activity on the tonoplast than that of salt-sensitive *Plantago media* plants, suggesting that the innate difference in the ability for the Na^+^ sequestration into vacuoles could be the cause of the difference in salt sensitivity (Staal et al. [1991]). Therefore, important questions to be addressed are whether salt tolerant cultivars of glycophytes or halophytes retain better systems such as superior enzyme activity including more preferable activity/ion selectivity of transporting proteins and more efficient regulations on the genes/proteins involved.

### Components for the Na^+^ reabsorption from the xylem vessel

Na^+^ ions that reach the xylem by passing through barrier mechanisms in roots under salinity stress are transported to shoots. In addition to independent barrier components introduced above, plants retain a different protection mechanism at the cell-xylem apoplast interface. It has been shown that Na^+^ reabsorption occurs from the xylem stream by surrounding tissues, and as a result, reduces the net Na^+^ flow into shoots (Läuchli [1984]; Lacan and Durand [1996]).

The *AtHKT1;1* cDNA that encodes a Na^+^ transporter has been isolated from *Arabidopsis* plants as a homolog of the *TaHKT2;1* gene, which is a Na^+^/K^+^ co-transporter in wheat (*Triticum aestivum*) (Schachtman and Schroeder [1994]; Rubio et al. [1995]; Uozumi et al. [2000]). The disruption of the *AtHKT1;1* gene was found to render the plants salt hypersensitive with the severe chlorosis in leaves as a result of more Na^+^ in leaves than the wild type plants (Mäser et al. [2002]). Several independent laboratories further demonstrated that AtHKT1;1 is an essential component to restrict a Na^+^ accumulation in shoots especially in leaves by controlling the net Na^+^ flow in the long distant Na^+^ transport via the stele (Berthomieu et al. [2003]; Sunarpi et al. [2005]; Davenport et al. [2007]; Horie et al. [2006]; Møller et al. [2009]). Particularly, Sunarpi et al. ([2005]) indicated using the anti-AtHKT1;1 peptide antibody that AtHKT1;1 localizes at the plasma membrane of xylem parenchyma cells known as a key cell layer controlling the flux of water and solutes in the xylem stream, demonstrating that AtHKT1;1 is a crucial factor in Na^+^ reabsorption from the xylem vessel (Figure 4b). Supporting this, the enhancer trap-mediated targeting expression of AtHKT1;1 in root stellar cells increased an efficiency of the shoot Na^+^ exclusion and salt tolerance in *Arabidopsis* plants (Møller et al. [2009]). Recently, AtHKT1;1-mediated ion currents were characterized by patch clamp analyses using GFP-labeled root stelar cells from wild-type and *athkt1;1* mutant plants and the result provided biophysical evidence that AtHKT1;1 mediates passive Na^+^ channel transport (Xue et al. [2011]). Furthermore, independent *athkt1;1* mutant alleles caused a reduction in the K^+^ level in xylem vessels and shoots, which were inverted to the increased Na^+^ levels in the same tissues (Sunarpi et al. [2005]). Together, these results led to a hypothesis that Na^+^ exclusion mediated by Na^+^ channel activity of AtHKT1;1 from xylem vessels into xylem parenchyma cells indirectly stimulates K^+^ loading to xylem vessels (Sunarpi et al. [2005]) (Figure 4b). This hypothesis is consistent with the earlier physiological findings that Na^+^ reabsorption at the xylem occurs in exchange for K^+^ (Läuchli [1984]). This mechanism is ideal since plants can manage to not only prevent Na^+^ over-accumulation but also enhance K^+^ accumulation in shoots (Figure 4b), which increases a K^+^/Na^+^ ratio in shoots and most importantly in leaves during salinity stress (Horie et al. [2009]; Hauser and Horie [2010]).

Similar Na^+^ reabsorption mechanisms have been found in cereals such as rice and wheat based on genetic QTL analyses. In rice, the *OsHKT1;5* gene has been identified, based on the influence of the shoot K^+^ content (*SKC1*) locus on the K^+^ accumulation in xylem sap and shoots during salinity stress, as one of the primary genes causing a difference in salt tolerance between a tolerant indica cultivar Nona Bokra and a susceptible japonica cultivar (Ren et al. [2005]). The locus was also found to cause a significant increase in the Na^+^ accumulation in xylem vessels and shoots while K^+^ in the same tissues accumulated in the opposite manners (Ren et al. [2005]). Given that the product of the *OsHKT1;5* gene from the salt tolerant cultivar encoded a Na^+^ transporter that appears to exhibit a higher Na^+^ transport activity than that from sensitive cultivar, the importance of Na^+^ reabsorption from the xylem and the accompanied K^+^ homeostasis for salt tolerance of rice plants were proposed (Ren et al. [2005]). The OsHKT1;5-mediated salt tolerant mechanism in rice was later found to be similar to the AtHKT1;1-mediated Na^+^ reabsorption mechanism in *Arabidopsis* (Horie et al. [2009]; Hauser and Horie [2010]). In wheat, *T. aestivum*, an essential salt tolerant locus, *Kna1*, which maintains a high K^+^/Na^+^ ratio in shoots during salinity stress, had been identified (Gorham et al. [1987]). A more recent QTL analyses using durum wheat revealed that the *Nax2* locus that reduces Na^+^ transport from roots to leaf blades by restricting Na^+^ contents of xylem sap is homoeologous to the *Kna1* locus, which were eventually suggested to be the *HKT1;5* gene in wheat (Munns et al. [2003]; James et al. [2006]; Byrt et al. [2007]). Interestingly, wheat QTL analyses also highlighted the *Nax1* locus, which is another salt tolerant locus that shows very similar features to the *Nax2* locus (Munns et al. [2003]; James et al. [2006]). The *Nax1* locus has been suggested to encode the HKT1;4 transporter (Huang et al. [2006]), which is a close homolog of AtHKT1;1 and HKT1;5 transporters in rice and wheat, all of which are classified into the class I HKT transporter (Horie et al. [2009]; Hauser and Horie [2010]). Further details regarding HKT transporters were extensively reviewed elsewhere (Munns and Tester [2008]; Horie et al. [2009]; Hauser and Horie [2010]). Together, these findings strongly suggest that class I HKT transporter-mediated Na^+^ reabsorption at xylem parenchyma cells is a key component for plants to maintain a high K^+^/Na^+^ ratio in leaves, which result in salt tolerance of the plants during salinity stress.

In addition to xylem Na^+^ loading, the SOS1 transporter in *Arabidopsis* plants has been suggested to play a crucial role in Na^+^ retrieval from xylem vessels depending on the degree of salinity stress (Shi et al. [2002]). This function overlaps with the role of class I HKT transporters in the stele. However, after considering the thermodynamics of Na^+^ transport with the estimated pH level of the stele, SOS1-mediated Na^+^ retrieval was deemed to be less likely (Munns and Tester [2008]).

Based on the shoot Na^+^ content of barley plants under salinity stress, it has been suggested that salt tolerance is highly associated with the ability to restrict Na^+^ accumulation in shoots (Jeschke [1984]). This implies a potential predominant contribution of a similar Na^+^ reabsorption mechanism to salt tolerance of barley as found in *Arabidopsis*, rice and wheat plants. Interestingly, however, Shabala et al. ([2010]) recently demonstrated using barley cultivars with differences in their salt tolerance that the extent of salt tolerance of the tolerant cultivars investigated are not necessarily associated with Na^+^ accumulations in xylem sap, but rather related to a significantly higher K^+^ loading property into the xylem stream, which maintain a higher K^+^/Na^+^ ratio in xylem sap and thus in shoots. Moreover, salt tolerant cultivars were found to exhibit more efficient Na^+^ sequestration in leaves than sensitive cultivars (Shabala et al. [2010]), suggesting that higher xylem K^+^ loading and Na^+^/H^+^ antiport activity in leaves could be more predominant mechanisms for barley plants to resist salinity stress. The molecular identity of the protein(s) that mediates K^+^ loading to the xylem apoplastic space under salinity stress is yet to be determined. However, K^+^ efflux activity mediated by the K^+^ outward-rectifying channel (KORC) and/or the nonselective outward-rectifying channel (NORC) (Wegner and Raschke [1994]; Wegner and De Boer [1997]) could be primary candidates for it (Horie et al. [2009]; Hauser and Horie [2010]).

In comparison with increasing evidence for the essentiality of the function of some HKT transporters at the cell-xylem apoplastic surface in salt tolerance, regulation mechanisms controlling their activity and expression are largely unknown. A combination of genetic analyses with high-throughput elemental profiling and DNA microarray-based bulk segregant analysis has revealed that the *AtHKT1;1* expression was controlled by the tandem repeat region, which is subsequently found to be a dense cluster of small RNAs, that localizes at approximately 4 to 5 kb upstream of *AtHKT1;1* (Rus et al. [2006]). A more recent molecular genetic approach demonstrated that a plant hormone cytokinin regulates AtHKT1;1-mediated Na^+^ accumulation in shoots through the transcription factors, ARR1 and ARR12, in *Arabidopsis* plants (Mason et al. [2010]). Further research focusing on the regulation mechanisms of xylem-parenchyma localized HKT transporters will be important to elucidate the contribution of Na^+^ reabsorption to plant salt tolerance.

### Toxic Na^+^ influx into roots

An increase in the Na^+^ concentration of the outer environment triggers passive Na^+^ influx into root cortical cells (Blumwald [2000]; Tester and Davenport [2003]). Net Na^+^ entry in plant roots is the consequence of passive Na^+^ influx and active Na^+^ efflux. The initial unidirectional Na^+^ influx into saline roots was found to be of a very high rate (Tester and Davenport [2003]). Several electrophysiological studies have indicated a primary role of voltage-independent (or weakly voltage-dependent) nonselective cation channels (VIC/NSCC) in such a flux of Na^+^ in saline conditions (see reviews: Amtmann and Sanders [1999]; Demidchik et al. [2002]; Tester and Davenport [2003]). VIC/NSCC currents were found in the cell/root of several plant species including cereals such as wheat (Tyerman et al. [1997]; Davenport and Tester [2000]) and barley (Amtmann et al. [1997]). Using the fluorescent sodium-sensitive dye, Kader and Lindberg ([2005]) suggested an involvement of NSCCs in Na^+^ influx into root cells of salt-stressed rice plants. Although the molecular identity of the protein mediating toxic Na^+^ influx is not yet known, cyclic nucleotide-gated channels and ionotropic glutamate receptor-like channels are the primary candidates (Demidchik et al. [2002]).

Other potential pathways for the toxic Na^+^ influx into roots are via K^+^ channels/transporters and HKT transporters. A characteristic feature of HKT transporters is the channel-like Na^+^ transport activity in the presence of a large amount of Na^+^ (Gassmann et al. [1996]; Xue et al. [2011]), which could contribute to Na^+^ over-accumulation in plants. Unlike the case of *Arabidopsis* plants, HKT transporters were found to form a gene family in cereals including rice (Garciadeblás et al. [2003]; Huang et al. [2008]). *TaHKT2;1*-knockdown wheat plants exhibit low Na^+^ influx phenotypes under salt stress and have reduced salt sensitivity (Laurie et al. [2002]). A Na^+^ transporter OsHKT2;1, one of the seven OsHKT transporters in a japonica rice cultivar Nipponbare, has been demonstrated to mediate Na^+^ influx into roots under K^+^-starved conditions (Horie et al. [2001]; Garciadeblás et al. [2003]; Horie et al. [2007]; Yao et al. [2010]). However, it was also demonstrated that the Na^+^ transport activity of OsHKT2;1 is rapidly down-regulated in intact rice roots even upon a mild salt stress, suggesting that the contribution of OsHKT2;1 to toxic Na^+^ influx during salinity stress is little (Horie et al. [2007]). Given that the OsHKT2;4 transporter exhibited a broad cation transport activity including Ca^2+^ in *Xenopus laevis* oocytes, and that immunological detection localized the OsHKT2;4 protein at the plasma membrane of rice root hair cells, a novel physiological role of OsHKT2;4 in Ca^2+^-linked processes has been proposed (Lan et al. [2010]). More recently, OsHKT2;4 was demonstrated to exhibit a strong K^+^ selectivity over divalent cations such as Ca^2+^ and Mg^2+^ with an atypical low Na^+^ transport activity compared to other HKT transporters (Horie et al. [2011c]), suggesting that the potential contribution of OsHKT2;4 to the toxic Na^+^ influx is unlikely.

Plants retain a large K^+^ transporter family, the KT/HAK/KUP transporter family (Gierth and Mäser [2007]; Ward et al. [2009]). It has been demonstrated that high Na^+^ concentrations inhibit K^+^ transport mediated by the HvHAK1 transporter from barley but triggers Na^+^ transport in yeast cells (Santa-María et al. [1997]). Moreover, it has been pointed out that even K^+^ channels that are highly selective for K^+^ over Na^+^ could mediate Na^+^ uptake at high salinity (Amtmann and Sanders [1999]). Na^+^ uptake analyses using rice root protoplasts revealed that K^+^-selective channels as well as NSCCs are involved in the toxic Na^+^ influx (Kader and Lindberg [2005]). Together, these results suggest that K^+^ transport systems might also contribute as entry sites for the toxic Na^+^ influx under salinity stress. Further research will be necessary to draw a complete picture of this important process related to plant salt tolerance.

### 3. Pathways of the radial solute movement and apoplastic transport barriers formed in roots

**Figure 5:**
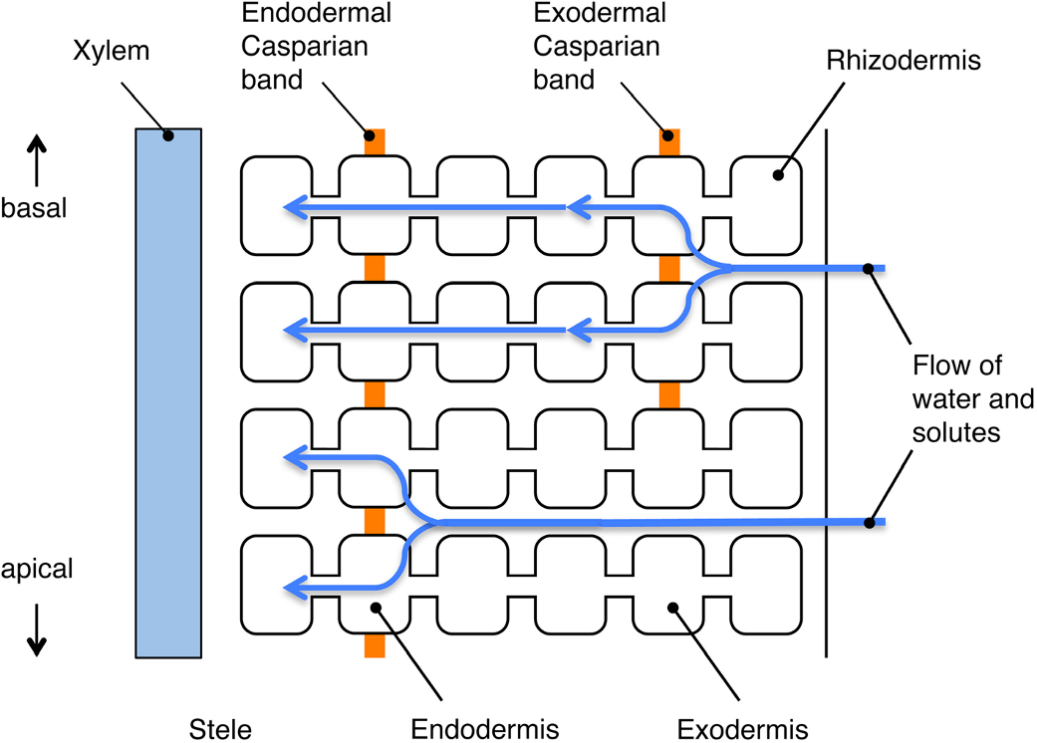
**Schematic diagram of a longitudinal section of a root illustrating general concept of radial movement of water and solutes.**

The root is the only organ that is directly exposed to excess salts under salt stress conditions, and at the same time, the root has important function to take up necessary solutes from the soil. Therefore, it is important how roots avoid the influx of excess salts. Solutes, once taken up by the roots from root surface, move across the root in the radial direction and enter xylem, where they are transported to the shoot. Potentially speaking, there are three pathways for the radial movement of solutes: apoplastic, symplastic and transcellular pathways (Figure 4a). Among these pathways, the transcellular component may be negligible because of the low membrane permeability of most solutes (Steudle and Peterson [1998]). A general understanding is that apoplastic transport barriers in roots are present in the endodermis and the exodermis (Perumalla and Peterson [1986]). The endodermis of roots of all vascular plants (Clarkson and Robards [1975]) and the exodermis of roots of many angiosperms (Perumalla and Peterson [1986]; Perumalla et al. [1990]) develop the Casparian band that is located in the transverse and the radial walls of cells of these tissues (Figure 5). Suberin is impregnated in the cell wall (Schreiber et al. [1999]; Zeier et al. [1999]) and the plasma membrane tightly attaches to the cell wall at the site of the Casparian band (Karahara and Shibaoka [1992]; [1994]). Suberin lamellae are formed in these tissues on the inner surface of the primary cell walls as secondary cell wall modifications after the formation of the Casparian band (Perumalla and Peterson [1986]). The function of the Casparian band is to avoid non-selective apoplastic radial movements of solutes into the stele, and the function of suberin lamellae is to block the passage of water and ions through the plasma membrane into the root stele (Steudle and Peterson [1998]; Schreiber et al. [1999]). Based on the earlier physiological and morphological studies, it is generally considered that the initial uptake of solutes could occur at the epidermis, at the exodermis, or if soil solution flows apoplastically across the root cortex, it would occur at the endodermis (Enstone et al. [2003]).

### Bypass flow of solutes and the apoplastic pathway in rice roots

From the general view point mentioned above, it is quite natural to hypothesize that the mechanism of salt exclusion could occur at the exodermis or at the endodermis in the radial direction, and from there, salt is transported radially via a symplastic pathway (Storey and Walker [1998]; Munns [2002]) because flow of solutes through the apoplast is arrested at the site of the Casparian band and finally solutes are selectively transported through the plasma membrane into the symplast (Figure 5). However, as described in the previous section, studies using apoplastic tracer showed that major pathway of uptaking Na^+^ ions into the stele is apoplastic pathway in the case of rice (Yeo et al. [1987]; Yadav et al. [1996]; Ochiai and Matoh [2002]).

As discussed by Garcia et al. ([1997]) and also mentioned in the previous section, the uptake of sodium ions by the bypass flow possibly involves direct apoplastic leakage occurring at the root apices (Enstone and Peterson [1992]) and the sites of secondary root emergence (Peterson et al. [1981]; Ranathunge et al. [2005]) (Figure 4a). On the other hand, experiments using pressure probes have indicated a possibility of some passage of water and perhaps also of solutes bypassing protoplasts (Steudle and Frensch [1996]). With regard to the outer part of the root (OPR), Ranathunge et al. ([2005]) performed experiments introducing precipitates of insoluble inorganic salts to block apoplastic pores and suggested existence of predominant apoplastic bypass flow of water as well as high permeability of ions of the OPR, supporting at least a possibility of apoplastic bypass flow of solutes in the OPR. Although it may appear somehow controversial about the mechanism of the bypass flow of solutes, a fascinating model called "composite transport" of both water and solutes is proposed. This model assumes that a switching between the apoplastic and cell-to-cell pathways occurs depending on given circumstances and explains how plants optimize water uptake according to demands from the shoot (Steudle and Frensch [1996]; Steudle and Peterson [1998]; Miyamoto et al. [2001]). The composite transport model may also be applied even to rice roots in which aerenchyma extensively develop (Ranathunge et al. [2005]).

A unique point of view accounting for the bypass flow discussed by Munns ([2002]) is that large gaps created between the plasma membrane and the cell wall may fill with solution and allow an artifactual apoplastic pathway for salts to move radially across the root under salinity stress conditions. However, this view might be unlikely at the sites of the Casparian band because plasma membrane tightly attaches to the cell wall at these sites even under osmotic (or salinity) stress conditions that causes severe plasmolysis (Karahara and Shibaoka [1992]).

Silicon alleviates abiotic stresses including salinity stress in rice (Matoh et al. [1986]; Ma and Yamaji [2006]). This function of silicon may be ascribed, in part, to the partial blockage of the transpirational bypass flow (Ma [2004]). In the case of alleviation of cadmium toxicity by silicon in maize, possible involvement of a change in the development of endodermal suberin lamellae is suggested (Vaculik et al. [2009]). Such a possibility could also be suggested in the case of alleviation of salinity stress by silicon in rice.

### Responses of roots with regard to the apoplastic transport barriers under salinity stress

To understand the detailed resistance mechanisms to salinity stress as well as of the bypass flow in rice roots, it is undoubtedly necessary to focus on the development and function of the apoplastic transport barriers in roots. Because an issue about the Casparian band development and its potential functions in salt tolerance has been recently reviewed (Chen et al. [2011]), here we refer mainly to rice roots. One of the most advantageous features of using rice for plant physiological studies is the availability of many cultivars (varieties) for comparative studies.

**Figure 6:**
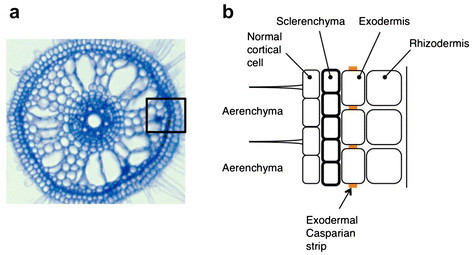
**Cellular compositions of the outer part of root (OPR) of a typical rice cultivar. (a)** Cross section of a four-day old root of *Oryza sativa* ssp. *japonica* cv. Nipponbare. The black rectangular corresponds to the area shown in **(b)**. **(b)** Schematic diagram of cellular compositions of a cross section of a root OPR.

Rice roots develop aerenchyma extensively in the cortex (Clark and Harris [1981]) and, after its formation, only several cell layers are left intact in the OPR. These cell layers in the OPR are composed of cortical cell layers that appear to be unmodified, sclerenchyma, exodermis, and rhizodermis from the inside to the outside (Clark and Harris [1981]) (Figure 6). It is suggested that low overall hydraulic conductivity of rice roots is a result of the existence of apoplastic barriers in the OPR and a strongly developed endodermis (Miyamoto et al. [2001]).

Among the structures in the OPR, a unique anatomical feature of rice roots, when compared with other species of cereal plants, is the formation of sclerenchyma of seminal roots and nodal roots (Galamay et al. [1991]) (Figure 6). This highly lignified tissue is formed so that it can presumably complement structural weakness caused by the extensive formation of aerenchyma in the cortex of rice roots. This sclerenchyma is composed of single or double cell layers and the number of this cell layers depends on rice varieties and has been considered to have a role in reducing water loss from roots (Feldman [1984]). However, no suberin could be detected in sclerenchyma by staining with Sudan Red 7B (Ranathunge et al. [2003]), indicating ineffectiveness of this tissue as an apoplastic barrier. Nevertheless, this sclerenchyma might be related to some unique physiological characteristics of rice roots, which is to be revealed in the future.

From the viewpoint of the issue about the bypass flow of water and ions, function of the OPR as the apoplastic barrier was examined using perfusion techniques (Ranathunge et al. [2003]; [2005]). Ranathunge et al. ([2005]) perfused the aerenchyma and the ORP with perfusion media containing different salts. When the salt of the outside of the root diffused across the apoplast of the OPR, the two different salts formed a coloured precipitate where they met, indicating a considerable permeability of the exodermal Casparian band to salts (Ranathunge et al. [2005]). This phenomenon may be attributed to a part of unique variable features of the exodermis (Hose et al. [2001]) and testing a permeability of the endodermis using the same technique would be interesting as mentioned by the same authors (Ranathunge et al. [2005]).

Chemical composition of the apoplastic barriers in roots is important for their functions (Schreiber et al. [1999]). In fact, the hydraulic conductivity of the apoplastic pathway can be decreased by the deposition of suberin in Casparian bands in the cell wall in maize roots (Zimmermann et al. [2000]). Suberin is a biopolymer consisting of an aliphatic and an aromatic domain with the aliphatic component being the major contributor to its apoplastic barrier functions (Schreiber et al. [1999]). To understand the importance of suberin in apoplastic barriers under salinity stress, comparative analyses of apoplastic barrier formation (i.e., morphology and suberin composition determined by gas chromatography and mass spectrometry) and Na^+^ uptake under salinity stress among different rice cultivars have been performed (Krishnamurthy et al. [2009]). They have found that suberization of apoplastic barriers in roots was most extensive in salt-tolerant cultivar, which also has the least sodium accumulation in the shoots. They also found that salinity stress induced the strengthening of these barriers in both sensitive and tolerant cultivars by increasing the expression of the genes encoding suberin biosynthetic enzymes. Cai et al. ([2011]) have also performed a similar comparative study using Fourier transform infrared spectroscopy and have found that development and deposition of suberin at the Casparian band in the endo- and exodermis were earlier, and the amounts of major chemical components in the OPR, such as aliphatic suberin, lignin and cell wall proteins and carbohydrates, were higher in the most salt-tolerant cultivar examined. These pieces of evidence indicate a correlation between development and suberization of apoplastic barriers and salt tolerance. Furthermore, chemical composition of suberin in rice roots and the roots hydraulic conductivity were analyzed and compared with those in maize roots by Schreiber et al. ([2005]). They have detected significantly higher amounts of suberin, i.e., 34 times greater in the endodermis and six times greater in the exodermis, in the apoplastic barriers of rice corresponded with a substantially lower root hydraulic conductivity compared with maize. It is no doubt that this higher amount of suberin content in the endodermis indicates the importance of the endodermis as an apoplastic barrier in rice roots. On the other hand, it is also suggested that this is true only for the endodermis because the OPR of rice root is highly porous and permeable to water and that, therefore, more detailed consideration of both the chemical nature and polymer arrangement of suberins in apoplastic barriers is necessary (Schreiber et al. [2005]; Ranathunge et al. [2011]). A similar opinion was also mentioned in the case of *Arabidopsis* roots based on a study using suberin mutants (Ranathunge and Schreiber [2011]).

Detailed morphological and developmental changes of the Casparian band under salinity stress have been studied in plant species other than rice. Morphometrical analysis performed at the ultrastructural level in maize roots demonstrated that the radial width of the Casparian band on a cross section of a root, a morphological parameter that should be related to the effectiveness of the Casparian band as an apoplastic barrier, increased under salinity stress, suggesting that the function of the band is enhanced under salinity stress (Karahara et al. [2004]). With regard to the development of the Casparian band, the distance from the root tip to the lowest position of the exodermal Casparian band decreased in cotton roots under salinity stress (Reinhardt and Rost [1995]). From these results, one may conclude salinity can accelerate the formation of Casparian band. It is, however, necessary to interpret these data carefully because the distance from the root tip to the lowest position of the Casparian band depends on cell division rate, cell elongation rate as well as the time required for formation of the band in individual cells. This issue was tested in the case of development of the endodermal Casparian band in maize roots under salinity stress and it was demonstrated that the estimated time required for formation of the endodermal Casparian band in an individual cell did not change under salinity stress even when the distance from the root tip to the lowest position of the Casparian band decreased (Karahara et al. [2004]). Therefore, a conclusion that Casparian band development is accelerated by salinity stress cannot be drawn exclusively from the observation that the band forms closer to the root tip under the stress. To solve this problem, a unique integrative analysis was proposed to monitor changes in the developmental processes of a particular cell type in the root, i.e. the rates of cell differentiation, production, and elongation (Karahara et al. [2008]). As a model case, effects of exogenous ethylene on differentiation, i.e. formation of the endodermal Casparian band, division and elongation of endodermal cells were analyzed in maize primary roots. Effects of environmental stresses, including salinity stress, on the development of barrier structures should be examined or re-examined by this approach. A recent finding of a novel protein family mediating Casparian band formation in the endodermis of *Arabidopsis* root (Roppolo et al. [2011]) has shed a new light on the study of the Casparian band development, which might also be relevant to salt sensitivity of plants.

## Conclusion

Soil salinity is a serious problem in the world agriculture. Owing to efforts of investigators and elevated levels of technologies, our knowledge on the mechanisms of plant salinity tolerance is dramatically expanding these days. However, individual plant species exhibits distinct salt sensitivity due to morphological differences and the difference in the ability of protection components that the plant has evolved to depend on. Therefore, challenges to elucidate the roles/significances of protection mechanisms in plant salt tolerance including morphological barriers at molecular, cellular and whole plant levels in further depth will be crucial to develop high-yielding salt-tolerant cultivars.

## Authors’ original submitted files for images

Below are the links to the authors’ original submitted files for images. Authors’ original file for figure 1 Authors’ original file for figure 2 Authors’ original file for figure 3 Authors’ original file for figure 4 Authors’ original file for figure 5 Authors’ original file for figure 6
