# Supplementary figures and images for: Salinity tolerance mechanisms in glycophytes: An overview with the central focus on rice plants
Source: Rice (N Y). 2012 Jun 22;5:11. doi: 10.1186/1939-8433-5-11 (PMC5520831; doi:10.1186/1939-8433-5-11)

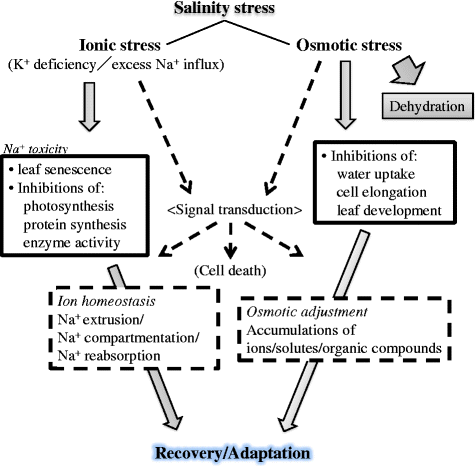

Supplement: Supplementary file 1 — Authors’ original file for figure 1 [file 12284_2012_9_MOESM1_ESM.gif]

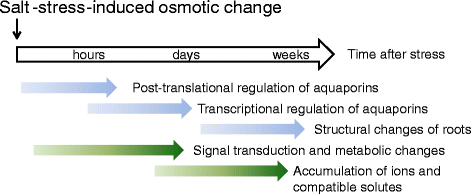

Supplement: Supplementary file 2 — Authors’ original file for figure 2 [file 12284_2012_9_MOESM2_ESM.gif]

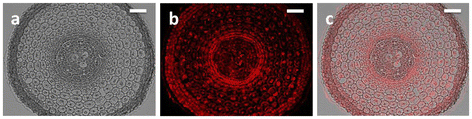

Supplement: Supplementary file 3 — Authors’ original file for figure 3 [file 12284_2012_9_MOESM3_ESM.gif]

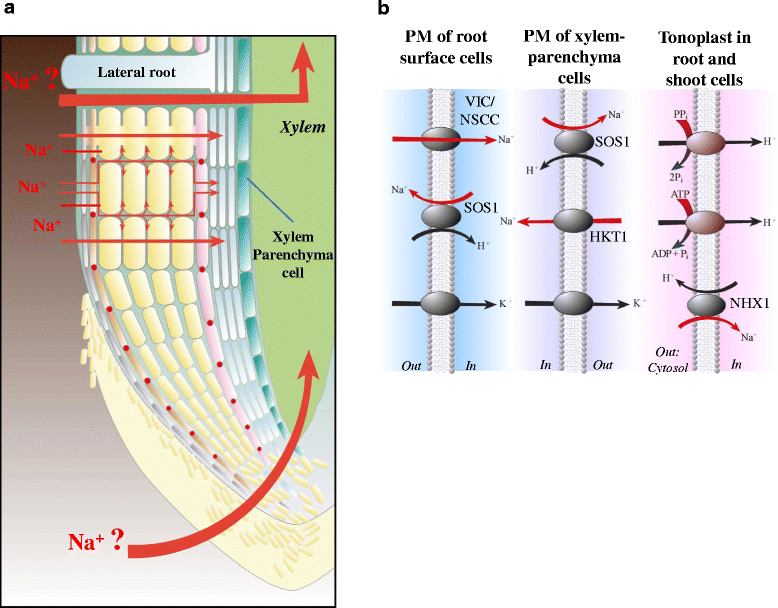

Supplement: Supplementary file 4 — Authors’ original file for figure 4 [file 12284_2012_9_MOESM4_ESM.gif]

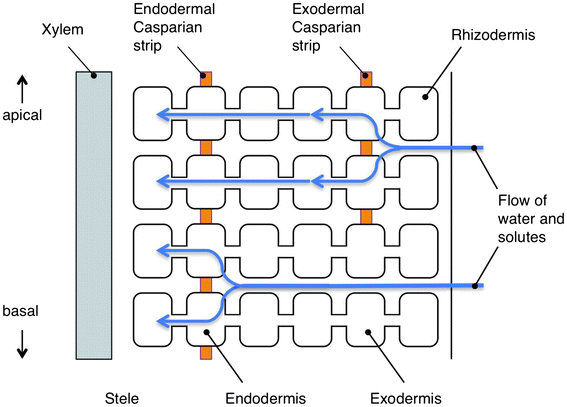

Supplement: Supplementary file 5 — Authors’ original file for figure 5 [file 12284_2012_9_MOESM5_ESM.gif]

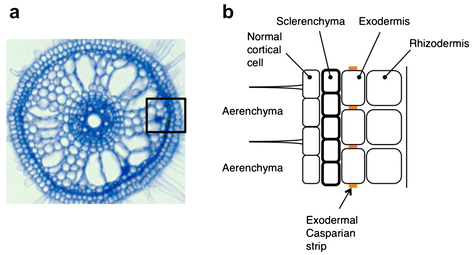

Supplement: Supplementary file 6 — Authors’ original file for figure 6 [file 12284_2012_9_MOESM6_ESM.gif]
